# Natural Polyphenols, 1,2,3,4,6-O-Pentagalloyglucose and Proanthocyanidins, as Broad-Spectrum Anticoronaviral Inhibitors Targeting Mpro and RdRp of SARS-CoV-2

**DOI:** 10.3390/biomedicines10051170

**Published:** 2022-05-18

**Authors:** Young-Hee Jin, Jihye Lee, Sangeun Jeon, Seungtaek Kim, Jung Sun Min, Sunoh Kwon

**Affiliations:** 1 KM Application Center, Korea Institute of Oriental Medicine, Daegu 41062, Korea; 2 Center for Convergent Research of Emerging Virus Infection, Korea Research Institute of Chemical Technology, Daejeon 34114, Korea; jsmin1019@kiom.re.kr; 3 Zoonotic Virus Laboratory, Institut Pasteur Korea, Seongnam 13488, Korea; jihye.lee_01@ip-korea.org (J.L.); sangeun.jeon@ip-korea.org (S.J.); seungtaek.kim@ip-korea.org (S.K.); 4 KM Convergence Research Division, Korea Institute of Oriental Medicine, Daejeon 34054, Korea

**Keywords:** COVID-19, SARS-CoV-2, SARS-CoV, MERS-CoV, natural polyphenol, 1,2,3,4,6-O-Pentagalloyglucose, proanthocyanidin, Mpro, RdRp, therapeutic

## Abstract

The natural plant dietary polyphenols 1,2,3,4,6-O-Pentagalloylglucose (PGG) and proanthocyanidin (PAC) have potent antioxidant activity and a variety of pharmacological activities, including antiviral activity. In this study, we examined the inhibitory effect of PGG and PAC on SARS-CoV-2 virus infection, and elucidated its mode of action. PGG and PAC have dose-dependent inhibitory activity against SARS-CoV-2 infection in Vero cells. PGG has a lower IC_50_ (15.02 ± 0.75 μM) than PAC (25.90 ± 0.81 μM), suggesting that PGG has better inhibitory activity against SARS-CoV-2 than PAC. The PGG and PAC inhibit similar Mpro activities in a protease activity assay, with IC_50_ values of 25–26 μM. The effects of PGG and PAC on the activity of the other essential SARS-CoV-2 viral protein, RdRp, were analyzed using a cell-based activity assay system. The activity of RdRp is inhibited by PGG and PAC, and PGG has a lower IC_50_ (5.098 ± 1.089 μM) than PAC (21.022 ± 1.202 μM), which is consistent with their inhibitory capacity of SARS-CoV-2 infection. PGG and PAC also inhibit infection by SARS-CoV and MERS-CoV. These data indicate that PGG and PAC may be candidate broad-spectrum anticoronaviral therapeutic agents, simultaneously targeting the Mpro and RdRp proteins of SARS-CoV-2.

## 1. Introduction

Coronaviruses (CoVs) are enveloped 30kb single-stranded positive-sense RNA viruses belonging to the Coronaviridae family [1]. Seven human CoVs (HCoVs) have been discovered. HCoV-229E, HCoV-OC43, HCoV-NL63, and HCoV-HKU1 usually cause mild respiratory tract infections and seasonal common cold symptoms, but severe acute respiratory syndrome CoV (SARS-CoV), SARS-CoV-2, and Middle East respiratory syndrome CoV (MERS-CoV) cause severe respiratory diseases, with high mortality rates. Since the SARS-CoV-2 outbreak started in December 2019, the World Health Organization (WHO) declared coronavirus disease 2019 (COVID-19) to be a pandemic in March 2020. As of April 2022, over 497 million confirmed cases of COVID-19 and over 6.1 million deaths have been reported globally [2]. Although over 11 billion COVID-19 vaccine doses have been administered, the current COVID-19 vaccines may not be efficient enough to prevent the COVID-19 pandemic, as new SARS-CoV-2 variants of concern, such as B.1.1.7 (or alpha), B.1.351 (or beta), P.1 (or gamma), and B.1.617.2 (or delta), continuously arise [3].

The SARS-CoV-2 genome shares 79% sequence identity with SARS-CoV, and 50% with MERS-CoV. Four structural proteins (spike proteins, envelope proteins, membrane proteins, and nucleocapsid proteins) and sixteen nonstructural proteins are encoded. Among them, the main protease, or 3C-like protease (Mpro or 3CLpro), a papain-like protease (PLpro), and an RNA-dependent RNA polymerase (RdRp) are essential for virus transcription and replication, and for potential targets for antiviral agents [4]. The sequence identity of Mpro in SARS-CoV-2 and SARS-CoV is approximately 96%, and SARS-CoV-2 RdRp has a 96% sequence identity to SARS-CoV RdRp, so resistance to Mpro or RdRp inhibitors in coronavirus variants is likely to be low [5,6]. SARS-CoV-2 Mpro and RdRp have a substrate specificity unique to the virus and are absent in humans, so a low toxicity is expected. The FDA approved the COVID-19 antiviral drugs Veklury (remdesivir) and Lagevrio (molnupiravir), which inhibit viral RdRp activity, and Paxlovid (nirmatrelvir/ritonavir), which inhibits Mpro activity, for the treatment of COVID-19 [7,8,9,10]. Although the therapeutic benefits are clear, these antiviral drugs have been reported to have serious adverse side effects. The side effects of Veklury include respiratory failure and organ dysfunction, and Lagevrio may cause fetal harm. Paxlovid is contraindicated with drugs which interact with CYP3A (e.g., α1-adrenoreceptor antagonists, HMG-CoA reductase inhibitors, antipsychotics, PDE5 inhibitors, sedatives, antimycobacterials, and anticonvulsants), and it may cause serious and life-threatening reactions or the loss of antiviral efficacy. Moreover, the development of more efficient broad-spectrum anticoronaviral therapeutics against new emerging coronavirus variants is still needed to overcome the COVID-19 pandemic and counteract new emerging coronaviral diseases.

Two types of dietary plant polyphenols, hydrolyzable tannins and condensed tannins, are abundant in fruits, red wine, and green tea. These compounds have strong antioxidant activity and various pharmacological activities; therefore, they have therapeutic potential [11,12]. A hydrolyzable tannin, 1,2,3,4,6-O-Pentagalloylglucose (1,2,3,4,6-penta-O-galloyl-beta-D-glucose, PGG), has been isolated from the peel of *Punica granatum* L. PGG has been reported to have antioxidant and antimutagenic activities through microarray expression profiling [13]. The anti-inflammatory activity of PGG is induced by inhibiting inducible nitric oxide synthase and cyclooxygenase-2 activity [14,15]. PGG is known to have antidiabetic activity [16], hepatoprotective functions [17], and a therapeutic effect in Alzheimer’s disease [18]. PGG has also been reported to have antiviral activity against HIV-1 [19], influenza A virus [20], hepatitis B virus (HBV) [21], and hepatitis C virus (HCV) [22]. PGG inhibits the rabies virus by suppressing the virus-induced miR-455-5p/SOCS3/STAT3/IL-6 pathway [23]. A condensed tannin, proanthocyanidin (PAC), has been isolated from the fruits of *Vitis vinifera* L. PAC has been shown to have antiapoptosis effects by ameliorating mitochondrial dysfunction [24]. PAC obtains its hepaprotective effect via CYP2E1 regulation [25]. The hypolipidemic [26] and cardioprotective activities [27] of PAC were previously reported. Additionally, it has been reported to exhibit antiviral activity against herpes simplex virus type 1 [28] and hepatitis C virus (HCV) [29].

In this study, we evaluated the broad-spectrum anticoronaviral activity of PGG and PAC against SARS-CoV-2, SARS-CoV, and MERS-CoV. To elucidate the mode of action of PGG and PAC, we examined their target viral proteins, such as Mpro, PLpro, and RdRp, using Mpro and PLpro protease activity assays, and a cell-based RdRp activity assay. This study identifies PGG and PAC as potentially valuable natural compound therapeutic candidates, which may inhibit emerging coronavirus infections, targeting two main viral proteins, Mpro and RdRp, simultaneously.

## 2. Materials and Methods

### 2.1. Test Compounds

**Figure 1:**
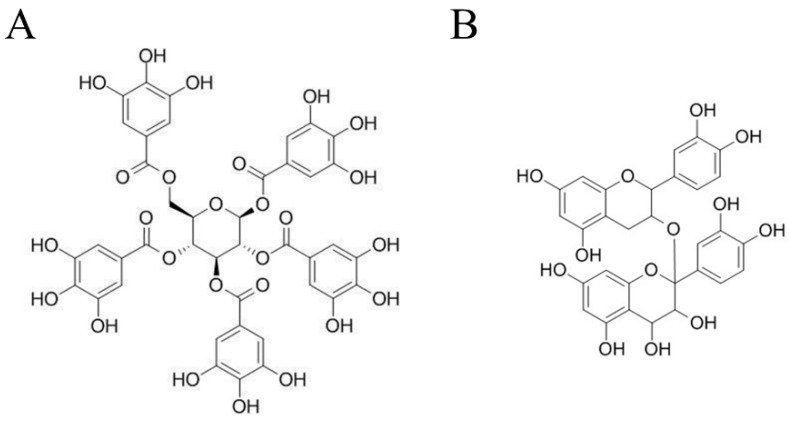
Chemical structure of 1,2,3,4,6-O-Pentagalloyglucose (PGG) (**A**) and Proanthocyanidins (PAC) (**B**).

1,2,3,4,6-O-Pentagalloylglucose (PubChem CID 65238, ≥98% purity) and proanthocyanidin (PubChem CID 122173182, ≥98% purity) were purchased from ChemFaces Biochemical Co. (Wuhan, China) (Figure 1). In total, 20mM stock solutions were prepared in dimethyl sulfoxide (DMSO; Sigma-Aldrich, St. Louis, MO, USA) and stored at −80 °C until use. Compounds were prepared to the indicated concentrations of up to 100 μM with fetal bovine serum-free medium before use. The concentration of DMSO in this experiment did not exceed 0.5%.

### 2.2. Cells and Viruses

Vero cells (CCL-81™) were purchased from the American Type Culture Collection (ATCC; Manassas, VA, USA) and cultured in Dulbecco’s modified Eagle’s medium (DMEM; Gibco, Carlsbad, CA, USA) with 1 × antibiotic–antimycotic solution (100 units/mL of penicillin, 100 μg/mL of streptomycin, and 250 ng/mL of amphotericin B; Gibco) and 10% fetal bovine serum (FBS; Gibco) in a 37 °C incubator under 5% CO_2_. The Korea Disease Control and Prevention Agency kindly provided SARS-CoV-2 (βCoV/KOR/KCDC03/2020) and MERS-CoV (MERS-CoV/KOR/KNIH/002_05_2015). SARS-CoV (strain HK39849) was kindly provided by Prof. J.S.M. Peiris from the University of Hong Kong. Virus propagation was performed using Vero cells. Experiments with coronavirus were conducted in a Biosafety Level 3 facility at the Institut Pasteur, Korea (IP-K; Gyeonggi, Korea), in compliance with the guidelines of the Korea National Institute of Health.

### 2.3. Immunofluorescence Antiviral Assays

Vero cells (1.2 × 10^4^ cells) were seeded in black 384-well culture plates (Corning Inc., Corning, NY, USA) containing DMEM, supplemented with 1× antibiotic–antimycotic solution (100 units/mL of penicillin, 100 μg/mL of streptomycin, and 250 ng/mL of amphotericin B, Gibco) and 2% FBS. After 24 h, the indicated concentration of compounds and SARS-CoV-2 (0.0125 multiplicity of infection, MOI), SARS-CoV (0.05 MOI), or MERS-CoV (0.0625 MOI) was added. After 24 h of incubation, the cells were fixed in 4% paraformaldehyde, and immuno-stained with anti-SARS-CoV-2 nucleocapsid protein antibody, anti-SARS-CoV spike protein antibody, or anti-MERS-CoV spike protein antibody (Sino Biological Inc., Beijing, China). Then, the cells were incubated with anti-rabbit IgG secondary antibody and Hoechst 33342 (Thermo Fisher Scientific, Waltham, MA, USA). The stained images were acquired using an Operetta^®^ High-Content Imaging System, and analyzed using Image-Mining 3.0 plug-in software (20×; PerkinElmer, Inc., Waltham, MA, USA), as previously described [30].

### 2.4. SARS-CoV-2 Mpro Activity Inhibition Assays

A purified maltose-binding protein (MBP)-tagged SARS-CoV-2 Mpro, a substrate peptide (14-mer FRET peptide, DABCYL-KTSAVLQSGFRKME-EDANS), and a serially diluted compound were incubated for 30 min, according to the manufacturer’s instructions (BPS Bioscience, San Diego, CA, USA). The fluorescence intensity was detected at an emission wavelength of 460 nm and an excitation wavelength of 360 nm using a Synergy H1 microplate reader (Bio-Tek, Winooski, VT, USA). GC376 (BPS Bioscience) was used as a positive control [31].

### 2.5. SARS-CoV-2 PLpro Activity Inhibition Assays

The enzymatic activity of SARS-CoV-2 PLpro was detected using SARS-CoV-2 PLpro assay kits (BPS Bioscience), according to the manufacturer’s instructions. The fluorescence intensity was measured at 360 nm/460 nm using a Synergy H1 microplate reader (Bio-Tek). GRL061 (BPS Bioscience) was used as a positive control.

### 2.6. Cell-Based SARS-CoV-2 RdRp Activity Assays

HEK293T cells (5 × 10^4^ cells, 30 passages) (CRL-11268, ATCC) were seeded in DMEM (Corning) supplemented with 10% FBS (Gibco) and 1% penicillin/streptomycin (Gibco) in a white 96-well plate (Corning). After 24 h, the RdRp expression plasmid, pCI-SARS-CoV-2 nsp12-N, and the reporter plasmid, p(+)FLuc-(−)UTR-NLuc, were transfected using transIT^®^-LT1 (Mirus Bio LLC., Madison, WI, USA), according to the manufacturer’s instructions. The serially diluted compounds were applied 6 h later, and the expression level values of Firefly luciferase (FLuc) and NanoLuc luciferase (NLuc) were measured using the Nano-Glo^®^ Dual-Luciferase^®^ Reporter Assay System (Promega, Madison, WI, USA) and Glomax (Promega) after another 18 h of incubation. To determine the activity of SARS-CoV2 RdRp, the expression of NLuc values was normalized with the expression of FLuc values, as previously described [32].

### 2.7. Statistical Analysis

Data were presented as the mean ± standard error of at least two independent experiments. The half-maximal inhibitory concentration (IC_50_) was calculated by nonlinear regression analysis using GraphPad Prism^®^ 9 (GraphPad Software Inc., San Diego, CA, USA). Probability (p) values were analyzed by one-way analysis of variance (ANOVA), followed by Bonferroni’s multiple comparison test using GraphPad Prism^®^ 9 (GraphPad Software Inc.), as indicated in the figure legends (* *p* < 0.05, ** *p* < 0.01, *** *p* < 0.001, and **** *p* < 0.0001).

## 3. Results

### 3.1. PGG and PAC Inhibited Infection with SARS-CoV-2

**Figure 2:**
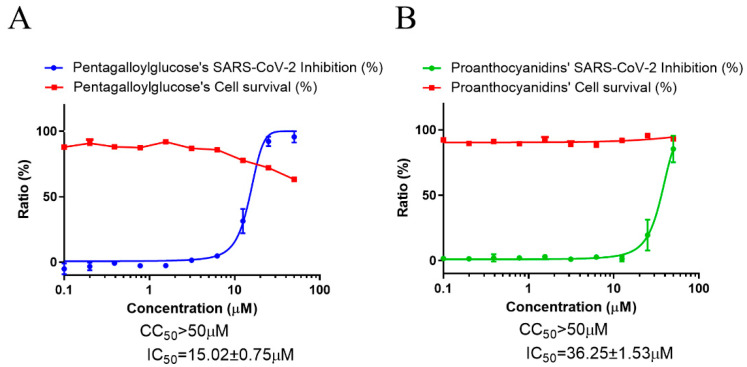
Anti-SARS-CoV-2 activity of PGG and PAC. (**A**) The dose–response curve analysis by the immunofluorescence-based antiviral assays showed the antiviral effect of PGG (**A**) and PAC (**B**) in SARS-CoV-2 infected Vero cells. Blue circles (**A**) and green circles (**B**) indicate the inhibition percentage of the SARS-CoV-2 infection by PGG and PAC, respectively, in a dose-dependent manner, and red squares indicate cell viability (%). The data represent duplicate experiments, and are presented as the mean ± SEM.

To examine the effect of PGG and PAC on SARS-CoV-2 infection, immunofluorescence-based antiviral assays were conducted using SARS-CoV-2-infected Vero cells treated with a serially diluted concentration of PGG or PAC and through detecting the SARS-CoV-2 nucleocapsid antigen after 24 h of infection. The data indicated that the CC_50_ values of PGG and PAC were >50 μM. PGG and PAC dose-dependently inhibited SARS-CoV-2 infection. The IC_50_ values of PGG and PAC were 15.02 ± 0.75 μM and 25.90 ± 0.81 μM, respectively (Figure 2). These data suggested that PGG and PAC had anti-SARS-CoV-2 activity, and that PGG had better inhibitory activity against SARS-CoV-2 than PAC.

### 3.2. PGG and PAC Inhibited the Mpro Activity of SARS-CoV-2

**Figure 3:**
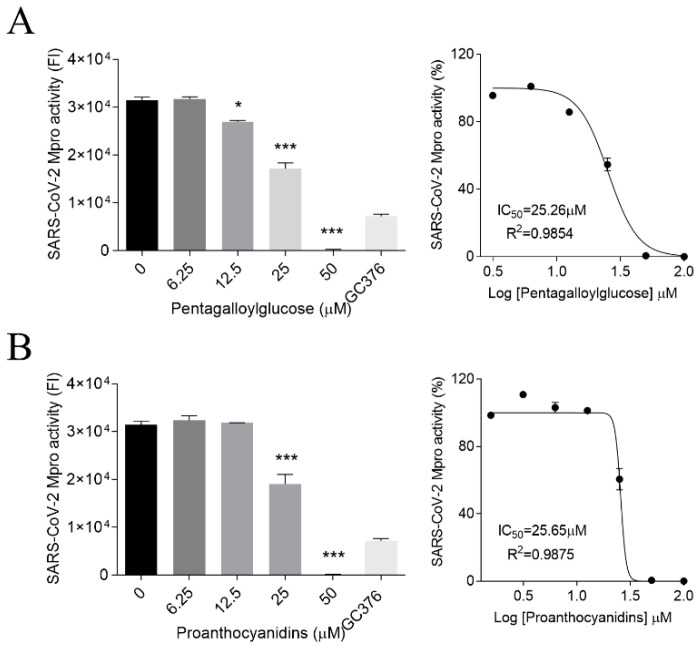
Inhibitory effect of PGG and PAC on the SARS-CoV-2 Mpro activity (**A**,**B**). PGG (**A**) and PAC (**B**) dose-dependently inhibited the Mpro activity of SARS-CoV-2 (left panel) (6.25~50 μM), and 100 μM GC376 was used as a positive control. The IC_50_ values are presented by nonlinear regression analysis (right panel). Statistical comparisons were conducted using one-way analysis of variance (ANOVA), followed by Bonferroni’s multiple comparison test. * *p* < 0.05; *** *p* < 0.001 vs. 0 μM. The data represent triplicate experiments, and are presented as the mean ± SEM.

To elucidate the mode of action of PGG and PAC, the effect of those compounds on the activity of the viral protease, Mpro, was examined using SARS-CoV-2 Mpro activity assay kits. When the Mpro protein was incubated with concentrations of PGG or PAC serially diluted from 100 μM, these compounds inhibited the Mpro activity in a dose-dependent manner (Figure 3). The IC_50_ values of PGG and PAC were 25.26 ± 1.04 μM and 26.00 ± 0.81 μM, respectively. These data showed that PGG and PAC inhibited the SARS-CoV-2 Mpro activity to similar extents. However, 25 μM of PGG and PAC did not inhibit the activity of another SARS-CoV-2 viral protease, PL protease, under our PLpro protein activity assay conditions (Figure S1).

### 3.3. PGG and PAC Inhibited the RdRp Activity of SARS-CoV-2

**Figure 4:**
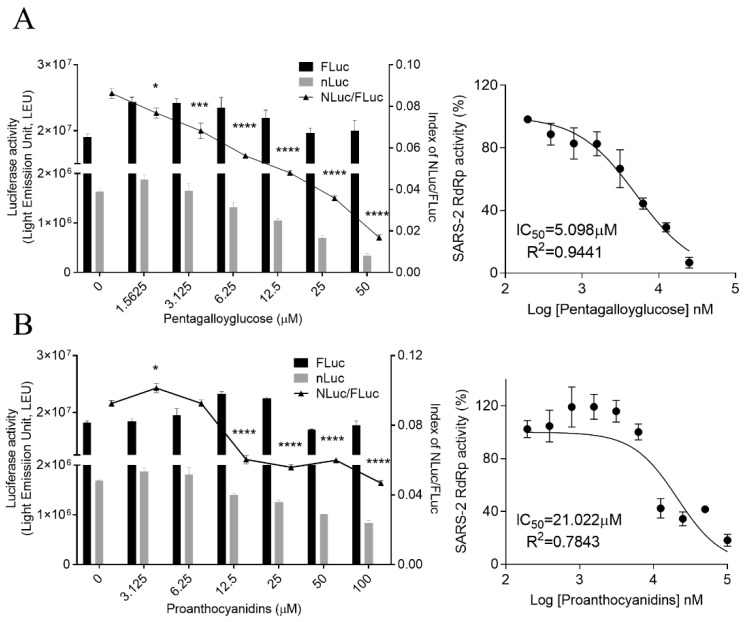
Inhibitory effect of PGG and PAC on the SARS-CoV-2 RdRp activity in the cell-based reporter assay system. A reporter plasmid, p(+)FLuc-(−)UTR-NLuc, and an RdRp expression plasmid, pCI-SARS2-nsp12N, were transfected in HEK293T cells. The cells were treated with serially diluted-PGG (**A**) and PAC (**B**) 6h later. After an 18 h incubation, the expression levels of FLuc and NLuc (left Y axis) were detected, and the NLuc/FLuc ratios (right Y axis) were calculated (left panel). Statistical comparisons were conducted using one-way analysis of variance (ANOVA), followed by Bonferroni’s multiple comparison test. * *p* < 0.05; *** *p* < 0.001; **** *p* < 0.0001 vs. 0 μM (left graph). The IC_50_ values were determined using nonlinear regression analysis (right graph). The data are representative of three independent experiments, and presented as mean ± SEM.

We also examined whether PGG and PAC could inhibit the activity of the essential viral protein, RdRp. We previously generated a cell-based SARS-CoV-2 RdRp activity assay system [32] and used it for determining the effect of PGG and PAC on the activity of RdRp using a treatment with serially diluted concentrations of PGG and PAC. In this system, the expression level of NLuc represented the activity of SARS-Co-2 RdRp, and the expression level of FLuc was used as the internal control to normalize NLuc activity, as previously described. The expression levels of NLuc were decreased in a dose-dependent manner through treatment with PGG or PAC. The levels of FLuc, the internal control, were maintained, suggesting that PGG and PAC inhibited the SRAS-CoV-2 RdRp activity without cytotoxicity or the inhibition of host transcription (Figure 4). Data showed that the IC_50_ values of PGG and PAC were 5.098 ± 1.089 μM and 21.022 ± 1.202 μM, respectively. These data suggest that PGG and PAC had inhibitory activity against the RdRp of SARS-CoV-2, and PGG had stronger inhibitory activity against the RdRp of SARS-CoV-2 than PAC. These findings were consistent with those that PGG had better inhibitory activity against SARS-CoV-2 than PAC, although these compounds had similar inhibitory activity against Mpro. Taken together, these results indicated that PGG and PAC inhibited SARS-CoV-2 infection by targeting the Mpro and RdRp of SARS-CoV-2.

### 3.4. PGG and PAC Inhibited Infection with SARS-CoV and MERS-CoV

**Figure 5:**
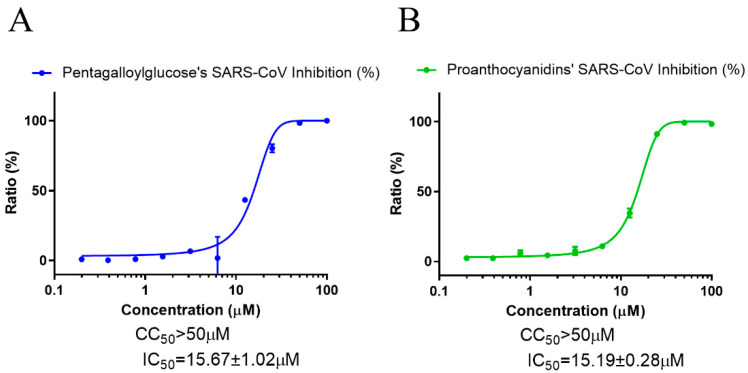
Anti-SARS-CoV activity of PGG and PAC. (**A**) The dose–response curve analysis using the immunofluorescence-based antiviral assays presented the antiviral effect of PGG (**A**) and PAC (**B**) on the SARS-CoV infection in Vero cells. Blue circles (**A**) and green circles (**B**) indicate the inhibition percentage of the SARS-CoV infection by PGG and PAC, respectively, in a dose-dependent manner. The data represent duplicate experiments, and are presented as the mean ± SEM.

We further examined whether PGG and PAC could inhibit other coronaviruses, SARS-CoV and MERS-CoV, to identify whether these compounds have broad-spectrum anticoronaviral effects. As in the SARS-CoV-2 experiment, immunofluorescence-based antiviral assays were conducted in 0.05 MOI SARS-CoV-infected Vero cells, treated with a serially diluted concentration of PGG or PAC, and the expression of the SARS-CoV spike protein was detected using an anti-SARS-CoV spike protein antibody, 24 h postinfection (Figure 5). The data showed that PGG and PAC dose-dependently inhibited SARS-CoV infection, with IC_50_ values of 15.67 ± 1.02 μM and IC_50_ 15.19 ± 0.28 μM, respectively, with a CC_50_ > 50 μM. These results suggested that PGG and PAC could inhibit the SARS-CoV infection in Vero cells to a similar degree.

**Figure 6:**
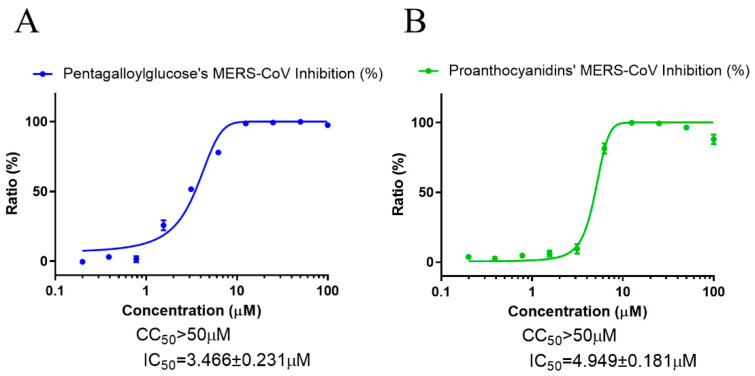
Anti-MERS-CoV activity of PGG and PAC. (**A**) The dose–response curve analysis using the immunofluorescence-based antiviral assays showed the antiviral effect of PGG (**A**) and PAC (**B**) on the MERS-CoV infection in Vero cells. Blue circles (**A**) and green circles (**B**) indicate the inhibition percentage of the MERS-CoV infection by PGG and PAC, respectively. The data represent duplicate experiments, and are presented as the mean ± SEM.

To assess the effect of PGG and PAC on MERS-CoV infection, immunofluorescence-based antiviral assays performed with 0.0625 MOI MERS-CoV-infected Vero cells were detected using anti-MERS-CoV spike protein, 24 h postinfection, and then analyzed. The data showed that PGG and PAC dose-dependently inhibited MERS-CoV infection, with IC_50_ values of 3.466 ± 0.231 μM and 4.949 ± 0.181 μM, respectively (Figure 6). Therefore, these data suggested that PGG and PAC could inhibit the coronaviruses SARS-CoV and MERS-CoV, as well as SARS-CoV-2.

## 4. Discussion

The polyphenolic natural products PGG and PAC are found in fruits, and exhibit strong antioxidant activity and a variety of pharmacological activities. The inhibitory effects of PGG and PAC on coronavirus infections were examined, and we found that PGG and PAC exhibited anticoronaviral activity against SARS-CoV and MERS-CoV, as well as SARS-CoV-2 in Vero cells, by simultaneously inhibiting the activity of Mpro and RdRp in SARS-CoV-2.

It has previously been reported that PGG blocks the entry of HCV [22] and human respiratory syncytial virus cell entry by using the pseudotyped lentiviral system [33]. Recently, the binding between PGG and the SARS-CoV-2 RBD protein has been shown using molecular docking and biolayer interferometry binding assays, and the blocking of SARS-CoV-2-RBD binding to hACE2 was shown by ELISA, immunocytochemistry assays, and spike protein RBD-pseudotyped lentivirus infection experiments [34]. PGG has also been reported to inhibit viral protein activity, such as HIV-1 integrase activity [19] and the activity of the neuraminidase of the influenza A virus [35]. Hydrolyzed tannins, including PGG, were shown to bind to Mpro proteins using surface plasmon resonance and molecular docking, as well as to inhibit the activity of Mpro in protein activity assays [36]. PAC was reported to inhibit SARS-CoV-2 replication by directly binding to the SARS-CoV-2-E channel [37]. The derivatives of flavan-3-ols, similar to PAC, procyanidin A2 (PA2), and procyanidin B2 (PB2), were shown to bind in the binding pocket of Mpro in docking simulation data. However, only PB2, not PA2, inhibited Mpro activity in Mpro protein activity assays [38]. In our data, PAC and PGG were compared and shown to inhibit Mpro activity. Overall, these data suggest that the presence of more hydroxy groups, galloylation, and the oligomerization of polyphenols such as PGG and PAC induce stronger inhibitory activity of Mpro [36], as it was previously discovered that higher numbers of hydroxyl groups of polyphenols produced enhanced antioxidant activity [11].

In this study, we demonstrated an inhibitory effect of PGG and PAC on SARS-CoV-2 infection in vitro. PGG had better inhibitory activity against SARS-CoV-2 infection than PAC. We also showed that PGG and PAC inhibited Mpro activity in protein activity assays to a similar extent, and that the activity of the other viral protease, PLpro, of SARS-CoV-2 was not inhibited by PGG or PAC under these experimental conditions. It was first reported that the activity of another essential viral protein, RdRp, was also dose-dependently inhibited by PGG and PAC, and PGG had a stronger inhibitory effect on SARS-CoV-2 RdRp activity than PAC. These observations suggested that the better inhibitory activity of PGG against SARS-CoV-2 was due to the better inhibition of the RdRp activity, despite its similar inhibitory activity against Mpro. We also demonstrated that PGG and PAC inhibited the other coronaviruses, SARS-CoV and MERS-CoV, suggesting that PGG and PAC have broad-spectrum anticoronaviral activity, which is particularly important in light of the ongoing emergence of coronavirus variants. However, the IC_50_ of PGG and PAC was much higher than those of the FDA-approved COVID-19 antiviral drug. Toxicity studies of PGG and PAC should be performed with the effective concentrations to exert an antiviral effect in the blood. Furthermore, we need to evaluate the proof of concept of PGG and PAC in in vivo experiments, pharmacokinetic properties (microsomal stability, hERG inhibition, CYP450 inhibition, etc.), and the potential transition to human trials, or the antiviral hygienic applications of PGG and PAC.

## 5. Conclusions

These findings suggest that PGG and PAC might be effective broad-spectrum anticoronaviral therapeutic candidates. The mode of action of PGG and PAC by targeting the Mpro and RdRp viral proteins of SARS-CoV-2 could provide valuable information for the development of therapeutic candidates against emerging coronaviral infections.

## Data Availability

Not applicable.

## Supplementary Materials

The following supporting information can be downloaded at: https://www.mdpi.com/article/10.3390/biomedicines10051170/s1, Figure S1: The effect of PGG and PAC on the SARS-CoV-2 PLpro activity. PGG and PAC (25 μM) did not affect the PLpro activity of SARS-CoV-2. GRL061 was used as a positive control.
