# Supplementary material for: Natural Polyphenols, 1,2,3,4,6-O-Pentagalloyglucose and Proanthocyanidins, as Broad-Spectrum Anticoronaviral Inhibitors Targeting Mpro and RdRp of SARS-CoV-2
Source: Biomedicines. 2022 May 18;10(5):1170. doi: 10.3390/biomedicines10051170 (PMC9138959; doi:10.3390/biomedicines10051170)
Supplement: Supplementary file 1 [file biomedicines-10-01170-s001.zip › biomedicines-1711008-supplementary.pdf]

Figure S1

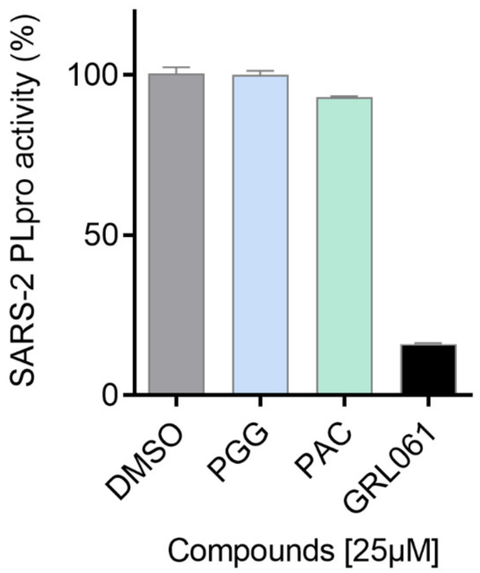

Figure S1. The effect of PGG and PAC on the SARS-CoV-2 PLpro activity. PGG and PAC (25 µM) did not affect the PLpro activity of SARS-CoV-2. GRL061 was used as a positive control.
